# Protein Intake, Physical Performance and Body Composition in Master Athletes—A Short Scoping Review

**DOI:** 10.3390/nu17030498

**Published:** 2025-01-29

**Authors:** Bernhard Franzke, Renate Maierhofer, Peter Putz

**Affiliations:** Research Center Health Sciences, FH Campus Wien University of Applied Sciences, 1100 Vienna, Austria; renate.maierhofer@fh-campuswien.ac.at (R.M.); peter.putz@fh-campuswien.ac.at (P.P.)

**Keywords:** elderly athletes, diet, muscle strength, muscle quality, muscle mass, exercise performance

## Abstract

Sufficient protein intake has been shown to be advantageous for developing muscle mass, muscle strength, muscle quality, as well as for improving recovery from strenuous exercise, all of which are essential for athletic performance. Aging athletes, so-called master athletes, would benefit from evidence-based recommendations; however, studies investigating the role of their protein intake on muscle quality or performance are diverse and rare. Consequently, existing recommendations for this subpopulation of aging athletes are non-existent or speculative in nature. The aim of this short scoping review is to summarize available reports, identify common outcomes, and suggest future research directions. Literature research was carried out in PubMedMedline, SPORTDiscuss, and ScienceDirect without any restrictions regarding year of publication, type of research or sports discipline. Only observational and interventional studies with data on protein intake linked with body composition or performance outcomes were included for further analyses. We identified 12 suitable reports on master athletes with very diverse age-ranges, a broad variety of sports, and very heterogeneous outcome parameters. Seven studies investigated endurance athletes, four studies investigated multi-sports athletes, and only one study reported on strength-trained athletes. Average protein intake ranged between 1.0 and 1.9 g/kg/d. Within the few available studies, evidence tends to point towards a benefit of higher protein intakes for muscle mass and function; however, the low number of studies, combined with heterogeneity in study design and methods, limits their generalizability. Future studies are needed to build the evidence base for clear dietary recommendations respecting the specific needs of aging athletes.

## 1. Introduction

With an increasing pace of population aging, aging itself is affecting our society more and more. The World Health Organization expects that the number of people aged over 60 will double between 2015 and 2050 [1]. With the prospective increase in the proportion of elderly people, an increase in most chronic diseases seems inevitable [2].

Parallel to the growing proportion of elderly humans, the number of master athletes, aged 35 years and older, is expected to rise [3]. Master athletes, in the literature often synonymously referred to as senior athletes, demonstrate, in many health-related aspects, superior outcomes compared to their peers, such as better bone mineral density, better cardiovascular health, sustained muscular function and lower levels of inflammatory markers [4,5,6]. Regular exercise is crucial for overall health [7], which seems to be a primary driver for master athletes successful aging model [8,9]. Specifically in the context of an (master) athlete’s performance, a sufficient dietary intake of all necessary nutrients is required [10]. Notably, in the context of aging and exercise (e.g., the aging athlete), prioritizing protein is required to optimize performance, recovery and health [11]. In general, aging goes hand-in-hand with a decline in muscle mass and physical function. Starting around 30 years of age, muscle mass and strength decline at a pace of around 10% per decade [12]. Another phenomenon observed with aging is the so-called anabolic resistance. Anabolic resistance is characterized by a higher threshold of skeletal muscle to anabolic stimuli, such as resistance exercise or dietary protein intake [13]. Regular practice of muscle stimulating exercise, together with an increased protein intake (~0.4 g/kg/meal, or <1.2 g/kg/d), could at least partly prevent or even reverse the occurrence of anabolic resistance [14]. Official protein intake recommendations for the physically active elderly (1.0 to 1.5 g/kg/d) [14] are being criticized by expert groups who are claiming benefits of much higher intakes (1.4 to 2.0 g/kg/d) [11]. In support of this, higher protein intakes showed superior outcomes for developing lean body mass [15,16] and muscle strength [17] in the elderly. These strategies are established and well researched in elderly non-athletic populations. The dietary needs of athletes have also been broadly discussed, and evidence supporting benefits of a higher protein intake than the current RDA is growing further [11].

Master athletes, specifically those in advanced age-groups, combine the physiological requirements of age-associated declining function and catabolism with performance-orientated exercise practices, and therefore represent a unique fairly heterogeneous setting. Although there are reports summarizing the general dietary habits of master athletes [18] as well as the benefit of higher protein intakes than the current RDA for physical function in the general aging population [19], current literature does not provide sufficient evidence to develop dietary guidelines, specifically for the crucial nutrient protein for aging athletes.

Therefore, the aim of this short scoping review was to summarize available reports about the link between protein intake with muscle mass, strength, muscle quality or performance in master and senior athletes and identify gaps for future research.

## 2. Materials and Methods

**Figure 1:**
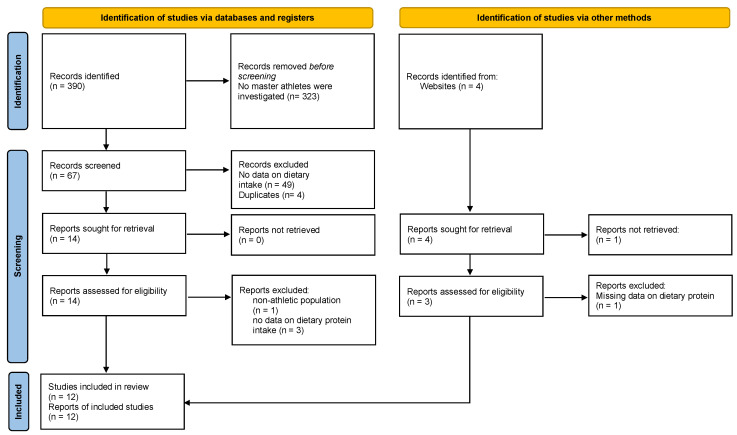
PRISMA flow diagram, (CC BY 4.0) (http://creativecommons.org/licenses/by/4.0/, accessed on 12 November 2024) [21].

The this short scoping review followed the PRISMA-ScR guidelines [20]. Literature search was performed until 13 January 2025. Database searches were run by Bernhard Franzke (1st author) in PubMed–Medline, SPORTDiscuss/EBSCO, and ScienceDirect/Elsevier; identification via other methods, such as ResearchGate.com and reference list screening, was performed independently by all authors. Literature research included the registers PubMed–Medline, SPORTDiscuss/EBSCO, and ScienceDirect/Elsevir. Using the search terms (‘master athletes’ OR ‘senior athletes’) AND (‘protein intake’) and without applying any filters, 390 records were identified through the database search; 4 additional records were available through ResearchGate.com. Figure 1 demonstrates the entire identification and selection process. Only human studies reporting original research (interventional or observational studies), without restrictions regarding publication time or publication language, were included in the final analysis. Reviews or meta-analyses were not included in this scoping review to prevent overlap of primary studies as a source of bias. Available reports needed to focus on master or senior athletes and needed to report data on dietary intake (protein) and body composition or performance related outcomes. Based on these criteria, 12 studies were included for further analysis. In this short scoping review, we use the term ‘master athletes’, and hereby also include the term ‘senior athletes’, which is also frequently used in the scientific literature.

## 3. Results

For this short scoping review, we were able to identify 12 reports suitable for further analyses. Included studies described master athlete populations from various sports backgrounds—see Table 1. The athletes’ ages ranged between 26 and 75 years. In total, 476 master athletes were part of the 12 studies identified for this short scoping review. Of 139 participants, only 29% were female master athletes.

Seven studies examined master athletes with an endurance-based background (triathlon, running, swimming) [22,23,24,25,26,27,28]; four studies reported on athletes from multi-sport backgrounds [17,29,30,31]; and one study presented data from strength trained master athletes [32].

Six cross-sectional studies [17,22,29,30,31] and one before–after study [27] reported daily protein intakes, ranging between 1.0 and 1.9 g/kg/d. Dietary intake was assessed through single or multi-days dietary recalls—one study used Food-Frequency Questionnaires [30]. These studies, with data on protein consumption, reported numbers of participants ranging between 8 and 176 [30].

The number of participants from the four identified intervention studies ranged between 5 and 24 master athletes [23,25,27,28]. Two studies focused on muscle protein synthesis rate and compared master athletes with age-matched controls [27] and younger athletes [23]. The only identified crossover study investigated the effect of high versus low post-exercise protein intake on recovery [25]. Nacleiro et al. [28] examined the effect of different protein sources on performance, body composition and blood biomarkers in 24 triathlon master athletes.

One study investigated the effect of high versus low protein intake on muscle mass and strength in master athletes. Girolamo et al. [17] observed that higher protein intake was linked to greater muscle strength in 50 elite master athletes.

In 58 master marathon athletes, Methenitis et al. [26] found that a higher protein intake was linked to favorable changes in body composition and metabolic blood biomarkers after a marathon race.

Sallinen et al. [32] showed that master athletes had greater muscle strength and muscle quality compared to controls, although their dietary intake was the same. In contrast, Hallfrisch et al. [29] observed that master athletes consumed significantly more protein and had less bodyfat compared to BMI- and age-matched controls.

Doering et al. [20] reported similar post-exercise protein intakes between older (58 ± 7 years) and younger (24 ± 4 years) triathletes. Notably, doubling the recommended post-exercise protein intake from 0.3 g/kg BW to 0.6 g/kg BW did not improve recovery, measured by knee extension peak torque and cycling time trial, but had beneficial effects on subjectively perceived fatigue recovery [25].

Within the 12 available studies, the average protein intake of master athletes ranged between 1.0 and 1.9 g/kg/d, thus being at or slightly below recommendations for sports nutrition (1.4–2.0 g/kg/d) or aging populations (1.0–1.5 g/kg/d) [14]. Beyond this, no clear pattern could be observed. The studies’ characteristics and main findings are summarized in Table 1.

**Table 1:** Characteristics and main findings of included studies.

First Author, Year, Reference	Study Type	Aim	Sport(s)	Age	Number (*n*) Gender (M/F)	Body Composition	Dietary Assessment/Protocol	Additional Relevant Methods	Main Outcome	Available Protein Intake in Masters (g/kg/d)
De Souza et al. (2024) [22]	Cross-sectional	Analyze timing, quantity, and source of dietary protein in competitive master athletes according to current recommendations	Swimming	48 ± 10 years	21 (21/0)	dual-energy X-ray absorptiometry	7-day food records	n.a.	Master swimmers presented a total protein intake within the recommendations for a daily basis. The majority of intake was at lunch and dinner.	1.9 ± 0.5 g/kg
Doering et al. (2016) [24]	Cross-sectional	Compare typical post-exercise intakes of carbohydrates and protein between masters and younger triathletes	Triathlon	Masters 58 ± 7 years Young 24 ± 4 years	Masters *n* = 51 (34/17) Young *n* = 30 (11/19)	n.a.	Representative dietary recalls	n.a.	Post-exercise protein intakes were similar between masters and younger triathletes. Relative to body mass, master triathletes (0.3 ± 0.2 g/kg) consumed significantly less protein than younger triathletes (0.4 ± 0.2 g/kg; *p* = 0.03)	n.a.
Girolamo et al. (2017) [17]	Cross-sectional	Test the hypothesis that protein intake level is associated with muscle strength in elderly elite athletes	Multi-sport	Low protein age = 72 years High protein age = 71 years	50 (38/12)	bioimpedance	Protein intake according to the median value of their ratio of urinary urea nitrogen to urinary creatinine, confirmed by dietary recall/interview	Muscle strength and muscle quality	A higher protein intake in elite senior athletes was associated with greater muscle strength and quality (r = 0.36; *p* = 0.01).	1.27 ± 0.25 g/kg
Hallfrisch et al. (1994) [29]	Cross-sectional	Diet and body composition were compared between master athletes and age- and BMl-matched sedentary men	Multi-sport	58–75 years	Masters: 16 (16/0) Age- and BMI-matched controls: 24 (24/0)	caliper and hydrodensitometry	7-day food records	n.a.	Master athletes showed higher protein intake (1.5 ± 0.11 g/kg vs. 1.2 ± 0.05 g/kg, *p* < 0.05) and lower body fat (%) (22.1 ± 1.4% vs. 26.4 ± 0.6%, *p* < 0.05) compared to age- and BMI-matched controls.	1.5 ± 0.11 g/kg
Leonhardt et al. (2024) [31]	Cross-sectional	Evaluate the dietary intake of highly competitive master athletes during world athletics championship competitions	Endurance, sprinting, jumping, multi-component, throwing	59 ± 10	43 (27/16)	n.a.	24 h recall		Protein intake was below the recommended levels for master athletes (1.5 g/kg), except for female athletes involved in power events (1.9 ± 0.1 g/kg)	1.3 ± 0.6 g/kg
Methenitis et al. (2021) [26]	Cross-sectional	Investigate the link between protein intake, marathon performance, body composition, acute race-induced changes, selected metabolic- and muscle damage-related biomarkers in recreational master runners	Marathon	58 ± 1 years	58 (58/0)	7 sites, skinfold calipers	3-day food records	Metabolic blood biomarker	Changes in body composition and metabolic indices were highly related to protein intake, either during the tapering period or during the race, with runners experiencing the lowest changes when consuming higher protein intakes	n.a.
Sallinen et al. (2008) [32]	Cross-sectional	Compare muscle strength and thickness, body composition, and dietary intake between master strength athletes and controls	Strength-trained athletes	young control men (26 years); middle-aged master athletes (52 years); middle-aged control men (52 years); older master athletes (72 years); older control men (71 years)	48 (48/0) young control men (*n* = 10); middle-aged master athletes (*n* = 9); middle-aged control men (*n* = 11); older master athletes (*n* = 8); older control men (*n* = 10)	caliper	4-day dietary recalls	Muscle strength and muscle thickness (ultrasound)	Master athletes showed greater strength and muscle quality compared to age-matched controls (52 y: *p* < 0.001, 72 y: *p* < 0.05); dietary intake did not differ between masters and control	52 y: 1.2 ± 0.3 g/kg 72 y: 1.0 ± 0.3 g/kg
Stanzione et al. (2022) [30]	Cross-sectional	Identify the average protein intake in g/kg LBM in a group of healthy Master Athletes	Multi-sport	39 years (26 years of age and older)	176 (82/94)	dual-energy X-ray absorptiometry	2005 Block’s Food Frequency Questionnaire (FFQ)	n.a.	Protein intake 1.43 ± 0.53 g/kg LBM; no gender difference (women 1.49 ± 0.53 g/kg LBM, men 1.36 ± 0.53 g/kg LBM, *p* = 0.12)	1.0 ± 0.4 g/kg
Doering et al. (2016) [23]	Before–after	Compare muscle protein synthesis (MPS) rates of master and younger triathletes over three consecutive days of intense endurance training	Triathlon	Masters (age, 53 ± 2 years) Young (age, 27 ± 2 years)	Masters *n* = 5 (5/0) Young *n* = 6 (6/0)	8 sites, skinfold calipers	3 d diet record before testing; individualized diets; all food consumed was recorded	muscle biopsies, MPS rate via deuterium-labeled water	Lower MPS rates in well-trained master triathletes over 3 d of training (*p* = 0.009)	n.a.
Doering et al. (2017) [25]	Crossover	Determine the effect of high post-exercise protein intake on recovery of knee extensor peak isometric torque, perceptions of recovery, and cycling time trial performance following EIMD in master triathletes	Triathlon	52 ± 2 years	8 (8/0)	n.a.	During recovery, a moderate or high protein intake (HPI) was consumed	Total Quality of Recovery (TQR; 6–20)	Doubling the recommended post-exercise protein intake from 3 × 0.3 to 3 × 0.6 g/kg did not improve recovery in master athletes; however, HPI provided moderate to large beneficial effects on perceived recovery/fatigue	n.a.
Mckendry et al. (2019) [27]	Before–after	Compare rested-state and exercise-induced rates of integrated myofibrillar protein synthesis (MPS) in endurance trained master athletes and healthy age-matched untrained individuals	Endurance-trained athletes	Controls 74 ± 3 years Master 69 ± 6 years	Controls *n* = 8 (8/0) Masters *n* = 7 (7/0)	bioimpedance	Standardized diet	muscle biopsies, MPS rate via deuterium-labeled water	Rested-state and resistance exercise-induced MPS were similar between master athletes and controls	1.3 ± 0.3 g/kg
Naclerio et al. (2017) [28]	Before–after	Compare effects of beef protein, whey protein, and carbohydrates on performance, body composition and blood biomarker	Triathlon	35–60 years old	24 (24/0)	air displacement, Bod Pod	Dietary recalls	vastus medialis muscle thicknesses (ultrasound); blood ferritin status	Beef protein beverage after workout or before breakfast (non-training days) can be effective in preserving thigh muscle mass and in improving iron status	n.a.

## 4. Discussion

The aim of this short scoping review was to summarize available reports about the link between protein intake with muscle mass, strength, muscle quality or performance in master athletes and identify gaps for future research. For our narrative synthesis, we identified 12 studies meeting these criteria; see Table 1. Protein intake in master athletes ranged between 1.0 and 1.9 g/kg/d. The International Society of Sports Nutrition recommends a protein intake from 1.4 to 2.0 g/kg/d to build or maintain muscle mass for exercising individuals. Regarding the elderly, they emphasize single dosages of 40 g would be necessary to optimize muscle protein synthesis [11]. Yet, specifics on the dietary protein intake of master athletes are missing in their current position regarding protein intake and exercise. From a broader perspective, with advanced aging, decline in muscle mass and function, together with a reduced adaptability of muscle following exercise or protein intake, needs to be addressed to reduce, stop, or reverse age-associated deterioration. In our own work, we were able to show that a higher protein intake of 1.6 g/kg/d, compared to 1.0 g/kg/d, significantly improved the adaptation to resistance exercise in elderly untrained individuals [16].

Expert groups, responsible for the formulation of dietary guidelines, recommend a protein intake between 1.0 and 1.5 g/kg/d for active elderly humans [14]. Several meta-analyses reported a beneficial effect of adequate and high protein intakes in the elderly on physical performance and muscle strength [33], the prevention of sarcopenia [34] and frailty risk [19]. These observations are also true for elderly women [35]. Yet, there are no evidence-based protein intake recommendations for master athletes.

Existing research on the optimized protein dosage and intake for exercise adaptation is inconsistent, but recent scientifically sound studies reported benefits of higher intakes than the current RDA. In their meta-regression, Morton et al. [36] conducted research on changes in lean body mass when combining resistance training and varying protein intakes. They reported a leveling-off of increases in muscle mass between 1.6 and 2.0 g/kg/d with no further benefit of higher intakes. Interestingly, two years later, Tagawa et al. [15] performed a similar comprehensive analysis with over 20 newly available studies and showed that slightly increasing current protein intake for several months by 0.1 g/kg/d in a dose-dependent manner over a range of doses from 0.5 to 3.5 g/kg/d may increase or maintain lean body mass. In support of the findings of Tagawa et al. [15], the latest research on protein dosage pointed to possible additional benefits of a single, very high instance of protein consumption (100 g) following an exercise stimulus [37]. However, independently of the recommendations, trials, or reports above, none of the presented data reported on master athletes, therefore leaving a broad research gap for this potentially growing population. Notably, if the benefits of higher protein dosages and intakes were also true for master athletes, specifically older age-groups, they might experience difficulties regarding digestibility and gastro-intestinal discomfort with such dietary modifications.

Currently, data on protein intake in master athletes is rare; see Table 1. Comparable to the findings above, the few available reports suggest a beneficial effect of a higher protein intake in this group, showing associations with greater muscle strength [17] and favorable body composition [29] compared to lower protein intake or age-matched controls, respectively. Further, higher protein intakes were associated with better stability of body composition and metabolic biomarkers in marathon runners post-race [26] and seemed to have positive effects on perceived exhaustion and fatigue following exercise induced muscle damage [25]. However, these observations require more research to be substantiated.

Interestingly, research on protein intake in the context of exercise is typically carried out in strength, explosive and muscle building related sports [11]. Yet, within the 12 identified studies for this scoping review, we found seven studies on endurance athletes (triathletes, swimmers and runners), four studies on multi-sport athletes and only one study on strength-based sports. This again underlines the incomplete data availability of this specific sub-population of aging athletes. Typically, strength athletes seem to benefit the most from increasing dietary protein intake [11]; however, our analyses did not show greater protein intakes in the study that focused on strength master athletes [32].

Available research on the link between protein intake, performance and body composition in athletic populations including master and senior athletes suggests a benefit of higher protein intakes than recommended by most official societies. Notably, data in senior and master athletes supporting this is very rare and all inferences are consequently speculative in nature.

### 4.1. Limitations

The 12 identified studies were very diverse regarding the age-ranges of master athletes (between 26 and 75 years old), study designs (eight cross-sectional, three before–after, one crossover), number of participants (5 to 176), methods assessing body composition (dual-energy X-ray absorptiometry, hydrodensitometry, bioimpedance, caliper) and dietary assessments (24 h, 3-day or 7-day food records, dietary recalls, food frequency questionnaire).

The reported age ranges themselves generate a very diverse environment, with master athletes’ mean age ranging across these 12 studies between 39 and 72 years. It is well reported that, compared to younger cohorts, non-athletic elderly past the age of 60 years require different recovery and nutritional strategies to maintain health [5,14]. Another essential parameter influencing the quality and generalizability of a study is the number of subjects. Here, again, we observed very diverse conditions, with study participant numbers in cross-sectional studies which reported protein intakes being between 21 and 176 subjects. Notably, with only 29%, female athletes were heavily underrepresented in the available studies. These aspects, combined with the low number of identified studies and the diverse methods of assessing body composition and dietary protein intake, severely limit the generalizability of our observations but generate implications for future research. Finally, the databases used for literature search may not have identified all sources of (gray) literature. Still, we believe that with PubMed, ScienceDirect and SPORTDiscuss we were able to detect the most meaningful publications for this scoping review.

### 4.2. Future Perspectives

In many aspects, master athletes demonstrate characteristics of proposed successful aging. However, the ‘How’ and ‘What’ behind their success is poorly described in the available scientific literature, specifically regarding elderly athletes’ nutritional habits and needs.

After thoroughly analyzing available literature on master athletes’ protein intake, physical performance and body composition, we suggest future studies focusing on:

- cross-sectional studies to establish representative data on dietary intake in master athletes, for both men and women, in different sports and age ranges;
- randomized controlled trials focusing on elderly master athletes (over the age of 60 years) investigating different amounts of protein intake for optimum adaptation to exercise and recovery;
- randomized controlled trials comparing responses to different protein intakes between old (e.g., 60 to 70 years), very old (e.g., 70 to 80 years) and super old (e.g., 80+ years) athletes;
- randomized controlled trials investigating possible gender differences regarding optimum protein intake and physiological adaptations in elderly master athletes.

Taken together, there is a clear need for more research regarding the optimum protein intake for master athletes (a) for specific sports, (b) in the context of exercise performance, (c) to optimize adaptation and recovery, and (d) to maintain optimum health.
